# Never, Ever Make an Enemy… Out of an Anemone: Transcriptomic Comparison of Clownfish Hosting Sea Anemone Venoms

**DOI:** 10.3390/md20120730

**Published:** 2022-11-23

**Authors:** Alonso Delgado, Charlotte Benedict, Jason Macrander, Marymegan Daly

**Affiliations:** 1 Department of Evolution, Ecology and Organismal Biology, The Ohio State University, Columbus, OH 43210, USA; 2 Department of Biology, Florida Southern College, Lakeland, FL 33815, USA

**Keywords:** venoms, toxins, transcriptomics, hemolytic, symbiosis, Anthozoa, clownfish, Entacmaea, Stichodactyla, Heteractis

## Abstract

Sea anemones are predatory marine invertebrates and have diverse venom arsenals. Venom is integral to their biology, and is used in competition, defense, and feeding. Three lineages of sea anemones are known to have independently evolved symbiotic relationships with clownfish, however the evolutionary impact of this relationship on the venom composition of the host is still unknown. Here, we investigate the potential of this symbiotic relationship to shape the venom profiles of the sea anemones that host clownfish. We use transcriptomic data to identify differences and similarities in venom profiles of six sea anemone species, representing the three known clades of clownfish-hosting sea anemones. We recovered 1121 transcripts matching verified toxins across all species, and show that hemolytic and hemorrhagic toxins are consistently the most dominant and diverse toxins across all species examined. These results are consistent with the known biology of sea anemones, provide foundational data on venom diversity of these species, and allow for a review of existing hierarchical structures in venomic studies.

## 1. Introduction

Cnidaria is the oldest venomous metazoan lineage, exhibiting the greatest functional diversity in venom across animals [1]. Cnidarian venom is stored within glandular cells, as well as within the cnidarian-specific cells called nematocytes that produce microscopic venom delivery structures called nematocysts [2,3]. Nematocysts are a shared feature that unite and diagnose members of the phylum, with structural variation in nematocysts delimiting groups within Cnidaria [4,5]. Venom is an integral component to cnidarian biology as it is used for prey capture, defense, intraspecific aggression, and digestion [6,7,8]. Sea anemones are found in all marine habitats and have widespread ecological success, likely because of the diversity of their ecological and physiological strategies [9,10]. The diverse symbiotic, competitive, and predator-prey interactions of sea anemones certainly rely on venoms [11], although precise correspondence between venoms, ecology, and physiology has yet to be demonstrated (but see [12]). Among the most compelling of the interactions in which venoms play a key role are mutualisms: sea anemones partner with photosynthetic and chemosynthetic microbes (e.g., [13,14]), other marine invertebrates (crustaceans, sponges, gastropods: [15,16,17,18,19]) as well as vertebrates, including clownfish [20].

The clownfish-sea anemone symbiosis has been a model system for research into complex mutualistic relationships. In this mutualism, the clownfish live in, mate, and lay eggs within the area covered by the body and tentacles of the host sea anemone. In at least some of the partnerships, the sea anemone host receives protection from predators by the clownfish [21,22,23] and indirectly benefits through the ammonia provided by the clownfish to the photosymbiotic zooxanthellae inside its tissues [24,25]. Much of the research into this symbiosis has focused on the clownfish, which are all members of the subfamily Amphiprioninae, a clade of ~30 species of clownfishes that has rapidly diversified within the damselfish family Pomacentridae [26,27].

In contrast to clownfish, which represent a single radiation [26,27,28,29], at least three lineages of anemones have independently evolved symbiotic interactions with clownfish [30]. These lineages, designated Stichodactylina (*Cryptodendrum*, *Heteractis magnfica*, *Stichodactyla*, *Thalassianthus*), Heteractina (*Heteractis aurora*, *Hs. crispa*, *Hs. malu*, and *Macrodactyla doreensis*), and *Entacmaea quadricolor,* are all part of superfamily Actinioidea but are not one another’s closest relatives [30].

Although the precise role of venom is unknown, it likely plays a role throughout this mutualistic symbiosis [31]. The venom of host anemones is inferred to be the deterrent to clownfish predators, and thus responsible for at least some of the benefits the fish receive [20,32]. Host anemones do not typically envenomate clownfish while associating with(see for [33] review), however, clownfish are susceptible to the venom of their host species [34]. This interaction is mediated through adaptive behaviors [20] and may change across the lifecycle of the fish [35]. Skin coating on the fish has been implicated as a factor explaining the nematocysts firing supression [31,36,37,38], with at least some of the protective attributes acquired through acclimatization by the fish to its host [39] and others inferred to be innate to the fish [40]. Genome-wide scans of clownfish find positive selection in genes associated with polysaccharides that might provide mechanisms for chemical manipulation of the mucus coat and thus offer some protection against host envenomation [41].

The venom variation across lineages of host sea anemones are poorly known. There are clear differences in the toxicity of venoms from different clownfish-hosting anemones, at least with reference to their mode of action on a crustacean model [42]. Variation in toxin effects does not follow a clear pattern [42], which is unsurprising given the lack of evolutionary relatedness across host anemones [30]. Beyond the ways in which venom might shape this complex and charismatic symbiosis, this system is ripe for investigation into venom constituents that have pharmaceutical potential. This is one of the few instances of clearly coevolutionary interactions between a sea anemone and a vertebrate, which creates interesting potential for specializations for the musculo-nervous system of vertebrates. Evidence of this relevance of the historical connection between host sea anemones and their fish associates for molecules with specificity to other vertebrates comes from *Stichodactyla helianthus*, the source of the medically promising polypeptide Sea Anemone Toxin ShK [43], and a member of this group. 

We investigate the venom in the sea anemones that have co-evolved with clownfish through a comparison of the transcriptomes from clownfish-hosting anemone representatives of each of the three major clades of anemone hosts: *Entacmaea*; Stichodactylina, and Heteractina. We use transcriptomes because this data source is most widely available for sea anemones, allowing more nuanced comparison with previous studies and other species. We use these accounts of expressed diversity of putative venoms and their function to identify differences and similarities of the venom repertoire of clownfish hosts.

## 2. Results

### 2.1. Transcriptome Assemblies

We assembled 20 transcriptomes from six species spanning the three symbiotic lineages using the using the de novo assembly program Trinity v2.2 [44]; Stichodactylina: *Cryptodendrum adhaesivum* (2), *Stichodactyla haddoni* (1); *Heterodactyla hemprichii* (4); Heteractina: *Heteractis crispa* (3), *Macrodactyla doreensis* (4); Entacmaea: *Entacmaea quadricolor* (6). Specific assembly details for concatenated transcriptomes are reported in Table 1, with specifics outlined for each transcriptome in Supplementary Table S1. The number of transcripts across assemblies ranged from 196,433 (*S. haddoni*) to 1,198,081 (*E. quadricolor*), with a mean value of ~700,000. The inferred number of genes ranged from 150,054 (*S. haddoni*) to 581,957 (*E. quadricolor)*. All N50 scores for each species were >1000 bp, except for *E. quadricolor* (N50 = 545 bp). BUSCO (Benchmarking Universal Single Copy Orthologs) scores for species transcriptomes ranged from 83.6–98.3%.

**Figure 1:**
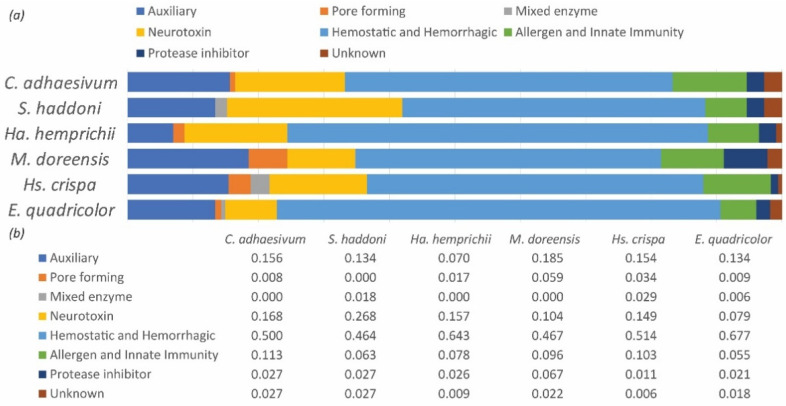
(**a**) Stacked bar graph showing the proportion of toxin functional catagories across each of the focal taxa. (**b**) table showing the percentage of toxin categories per species.

Transcripts with the presence of a signaling region and with high sequence similarity/bit scores and low E-value to known toxins from ToxProt were identified as candidates and extracted from each transcriptome assembly. Each of these were identified based on PFAM annotation, Tox Prot ID, and NCBI’s non redundant (NR) database BLAST results. These putative toxins were categorized into toxin families and then categorized into functional catagories: auxiliary toxins, allergens and innate immunity toxins, hemostatic and hemorrhagic activity toxins, mixed function enzymes, neurotoxins, pore forming toxins (cytolysins), protease inhibitors, and actiniarian toxins of unknown function. Proportion of functional venom catagories in the total recovered venom arsenal of each of our focal taxa was reported (Figure 1).

### 2.2. Putative Toxins Inferred from Transcriptomes across Clades

#### 2.2.1. Stichodactylina

We identified 483 putative toxins belonging to 47 toxin gene families (18 actiniarian-specific) from the members of Stichodactylina (*C. adhaesivum*, *Ha. hemprichii, S. haddoni*). The greatest diversity and number of putative toxins were recovered from *C. adhaesivum*, which had 256 putative venom transcripts from 34 toxin gene families in its transcriptome. The transcriptome of *C. adhaesivum* shows high representation of hemostatic and hemorrhagic toxins (128), auxiliary toxins (40), and neurotoxins (43) which together represent 81% of all putative toxin transcripts. In *C. adhaesivum*, we found 88 transcripts belonging to 13 actiniarian-specific toxin families, representing 37% of all transcripts. Among the anemone-specific toxin families, *C. adhaesivum* had a high representation of auxiliary toxin transcripts (39) and neurotoxins (33), which together comprised of 81% of the actiniarian-specific transcripts. *Cryptodendron adhaesivum* was the sole member of Stichodactylina in which we recover transcripts assigned to the actiniarian-specific toxins sea anemone 8 toxin and sea anemone structural class 9a toxins. We did not recover any mixed enzyme proteins (e.g., PLA2) from the transcriptome of *C. adhaesivum.*

The 115 putative toxin transcripts we recovered from *Ha. hemprichii* represent 32 toxin gene families. The transcriptome of *Ha. hemprichii* shows high representation of hemostatic and hemorrhagic toxins (74) and neurotoxins (18) which together represent 80% of all putative toxin transcripts. Although it contains close to the same number of actiniarian-specific gene families as the other stichodactylines, the transcriptome of *Ha. hemprichii* had the fewest number of actiniarian-specific toxin transcripts (27/115, 23%). The most abundant of these was the neurotoxin sea anemone type 3 potassium channel toxin (BDS). Despite the poor representation of putative toxin genes in the transcriptome assembly of *Ha. hemprichii*, this is the sole member of Stichodactylina for which we recovered a transcript for a cnidarian Small Cysteine-Rich Protein (SCRiP) and ShK-like-1 neurotoxin.

A total of 112 putative toxins belonging to 35 toxin gene families were identified from the transcriptome of *S. haddoni*. The transcriptome of *S. haddoni* shows high representation of hemostatic and hemorrhagic toxins (74) and toxins with neurotoxin activity (30), which together represent 82% of all putative toxin transcripts. From its transcriptome we identified 46 transcripts belonging to 12 actiniarian-specific toxin families, representing 38% of all toxin transcripts. As was the case for *C. adhaesivum*, *S. haddoni* showed strong representation in both the neurotoxin activity transcripts (25) and auxiliary activity transcripts (13), representing 82% of all putative actiniarian toxin transcripts. We did not recover a membrane active protein, actinoporin, or MAC/perforin domain protein from the transcriptome of *S. haddoni*.

#### 2.2.2. Heteractina

In our representative members of Heteractina, *Hs. crispa* and *M. doreensis*, we recovered 310 transcripts assigned to 37 toxin gene families, with 13 of these belonging to actiniarian-specific gene families. In the transcriptome of *Hs. crispa,* we recovered 175 putative toxin transcripts belonging to 35 gene families. The transcriptome of *Hs. crispa* was heavily represented by hemostatic and hemorrhagic toxins (90/175, 51%) which made up half of the total number of transcripts recovered. Transcripts identified as venom prothrombin activator (32), ryncolin (13), and veficolin-1 (10) were the most abundant hemostatic and hemorrhagic toxins. We identified 59 transcripts (34%) that belong to 12 actiniarian-specific gene families. Most common among these are auxiliary activity, astacin-like metalloprotease toxin M12A (13 transcripts) and nematocyst expressed protein, NEP-6 (13 transcripts), which together represent 54% of all putative actiniarian toxin transcripts. Among Heteractina, phospholipase A2 (PLA2) was found only in *Hs. crispa*, which showed more putative neurotoxin activity proteins, cysteine-rich venom proteins, and potassium channel toxins (type II & III) than *M. doreensis*.

We identified 135 putative toxin transcripts in the transcriptome assembly for *M. doreensis;* these belong to 25 toxin gene families. The transcriptome of *M. doreensis* is rich in hemostatic and hemorrhagic toxins (63/135, 46%) and auxiliary proteins (25/135, 18%), representing 64% of all transcripts recovered. Transcripts identified with venom protease (11) and Snaclec (10) gene families were the most represented transcripts among hemostatic and hemorrhagic toxins. We recovered 48 transcripts (38%) that were assigned to 12 actiniarian-specific gene families. The transcriptome of *M. doreensis* includes notable representation of transcripts identified as belonging to membrane active activity protein MAC/perforin domain (7 transcripts) and venom Kunitz-type protease inhibitor (9 transcripts) gene families. Among Heteractina, *M. doreensis*. was the only species to recover a transcript from the sea anemone 8 toxin family (1). We did not recover SCRiP, ShK-like-1 neurotoxin, sea anemone short toxin (type III), type II sodium channel toxins, or sea anemone structural class 9a toxins from the transcriptomes of Heteractina studied here.

#### 2.2.3. Entacmaea

*Entacmaea quadricolor* is the only member of *Entacmaea* to host clownfish. We found 328 putative toxin transcripts in its transcriptome, representing 37 toxin gene families. The transcriptome of *E. quadricolor* includes hemostatic and hemorrhagic toxins (222/328, 67%), with the true venom lectin (26), ryncolin (35), and veficolin-1 (45) families heavily represented. Of the 328 transcripts, 79 (24%) belonged to 13 actiniarian-specific gene families, with strong representation of the auxiliary activity proteins astacin-like metalloprotease toxin M12A (22) and nematocyst expressed protein NEP-6 (21). Despite having the second-highest number of actiniarian-specific putative toxin transcripts, we recovered no neurotoxins (including no transcripts similar to any voltage gated potassium or sodium toxins) in the transcriptome of *E. quadricolor*.

### 2.3. Diversity of Toxins within Functional Groups

A primary objective of this study was to identify functional groupings of toxins and characterize their distribution and diversity across actinioidean lineages that have independently associated with clownfish. We found multiple clade-specific putative toxins in Stichodactylina, and one unique putative toxin each in Heteractina and Entacmaea. Collectively, these unique putative toxins were low in abundance and in every functional class except mixed enzymes and pore-forming toxins. Additionally, knowing that the clownfish-hosting anemones represent independent associations in terms of the host lineage [30], we have looked at shared transcripts within a gene family (Table 2) and also at groups of functionally similar toxins that may include genes from many gene families to understand whether there are similarities in the composition of the venom arsenal at a functional level, if not at a phylogenetic one.

#### 2.3.1. Hemostatic and Hemorrhagic Toxins

Hemostatic and hemorrhagic toxins were the most diverse and prevalent types of putative venom genes recovered in this study. These toxins are associated with blood coagulation, inflammation, myotoxicity, platelet aggregation, and homeostasis interference and are ubiquitous in venom [48] and well documented among cnidarians [49,50,51,52].

Several hemostatic or hemorrhagic toxins were found across all studied taxa. We recovered abundant transcripts of hemotoxin venom prothrombin activator all species: *C. adhaesivum* (25), *Ha. hemprichii* (15), *S. haddoni* (5), *M. doreensis* (8), *Hs. crispa* (32), and *E. quadricolor* (48). Transcripts that closely match veficolin toxins that interfere in platelet aggregation [53] were recovered in all species, with the highest abundance in *E. quadricolor* (45). Transcripts matching the anti-coagulant ryncolin were found in all species and highly expressed in *E. quadricolor* (35). Hemorrhagic toxins similar to zinc metalloproteinase/disintegrins were recovered in all species. Peptide isomerase heavy chain transcripts were recovered across all species and heavily expressed in C. *adhaesivum* (17) and *E. quadricolor* (15).

We recovered transcripts matching American bumble bee venom protease activators [54,55] and Snaclec family, and c-type snake lectins [56] across all species. Transcripts similar to Snake venom lectins were recovered in all species but *Ha. hemprichii*. In all species but *M. doreensis,* we recovered transcripts closely matching blarina toxins (BLTX), which have dilatory effects on blood vessel walls in short tailed shrew [57]. Transcripts highly similar to snake venom coagulation factor V & X transcripts were also found in all taxa except *M. doreensis*. The procoagulant hemolin (EC 3.4.21.) toxin was not recovered in *S. haddoni* or *Hs. crispa*, but was found in *C. adhaesivum, E. quadricolor*, and *Ha. hemprichii.* Cobra venom factors (VF) from the venom complement C3 homolog family [58] were not recovered in *S. haddoni* or any members of Heteractina.

Putative toxin transcripts for snake venom coagulation factor V & X were recovered across all species except *M. doreensis.* We recovered transcripts with high similarity to two types of toad fish toxins: galactose-specific lectin nattectin [59,60], a pro-inflammatory activity toxin which also induces neutrophil mobilization, was recovered in all species but *Ha. hemprichi.* The proteolytic and myxotoxic toxin, natterin-4 [61] was recovered only in *Hs. crispa* (4) and *E. quadricolor* (1).

#### 2.3.2. Neurotoxins/Protease Inhibitors

Cnidarian neurotoxins are a set of diverse and well-characterized sodium and potassium channels toxins used to both immobilize the prey and to defend against predators [62]. They are widely used in the development of pharmaceuticals and bioinsecticides [63,64,65,66]. In this study, neurotoxins represented the second-most diverse type of toxins recovered in focal taxa. We found sea anemone type I and III potassium channel toxin subfamily (KTx1, KTx3) transcripts across all clades, with transcript abundances ranging from 1–9 (Table 3). The only species in which we found no KTx1 transcripts is *Ha. hemprichii.*

The cnidarian-specific neuropeptide SCRiP [8], originally described in reef building corals, was recovered in only *Ha. hemprichii* (1) and *E. quadricolor* (1). Cysteine-rich venom proteins were recovered in *C. adhaesivum* and in both members of Heteractina. Sea anemone structural class 9a transcripts were recovered in the stichodactylines *C. adhaesivum* (1) and *S. haddoni* (1) and in *E. quadricolor* (3).

In all species, we recovered transcripts matching to the conopeptide p-like superfamily. In cone snails, these proteins act both as neurotoxin and serine protease inhibitor [67]; they were found in high relative abundance in both *C. adhaesivum* (10) and *E. quadricolor* (8). We found transcripts closely matching neurotoxic u-scoloptoxin [68] in *S. haddoni* (1) and *Hs. crispa* (2).

Kunitz-domain peptides block ion channels and inhibit protease, leading to blood coagulation and inflammation [69]. Transcripts matching the venom Kunitz-type family were found across all species, with Heteractina member *M. doreensis* recovering the greatest number of transcripts (9). Sea anemone type II potassium channel toxin (KTx2) is a neurotoxin that has Kunitz domain; transcripts belonging to this group were abundant in all species, being most abundant in *C*. *adhaesivum* (11) and *E. quadricolor* (9).

Only in Stichodactylina did we recover transcripts identified as sea anemone sodium channel toxin type II (NaTx2). Both *S. haddoni* and *Ha. hemprichii* had transcripts matching the sea anemone short toxin (potassium type III) family. In *Ha. hemprichii*, we found the only ShK-like-1 neurotoxin transcript, similar to what has previously been described as type II from the model actiniarian *Nematostella vectensis* [8,70]. A transcript closely matching the protein omega-theraphotoxin-Pm1a was only recovered in *S. haddoni.* Additionally, a single transcript closely matching psalmotoxin-1 (PcTx1) was only recovered in *S. haddoni.* Psalmotoxins have pharmacological neuroprotection applications [71].

#### 2.3.3. Auxiliary Venom

Auxiliary toxin proteins act as venom stabilizers and are known to induce hemorrhaging and cause necrosis by degrading the extracellular matrix [72]. We found cnidarian-specific nematocyst expressed protein (NEP-6) transcripts, first characterized in the nematocysts of the starlet sea anemone, *Nematostella vectensis* [73], across all species. The greatest number of copies of NEP-6 were in *C. adhaesivum* (24) and *E. quadricolor* (21).

Across all species, we recovered astacin-like metalloprotease toxin (M12A). We recover transcripts closely matching the cysteine-type endopeptidase inhibitor cystatin-2 in all taxa except the stichodactylines *C. adhaesivum* and *Ha. hemprichii.*

*Crypotdendrum adhaesivum* was the only species in which we recovered auxiliary venom protein 302. Venom protein 302 has been recovered in many taxa, including the zoantharian cnidarians *Protopalythoa variabilis* and model actiniarian *Exaiptasia pallida* (see [49,74]). In *C. adhaesivum* we recovered a singular copy of venom protein 59.1, a protein associated with insulin-like activity [75]. We also found a reticulocalbin-2 in *Ha. hemprichii*, similar to a taipoxin-associated calcium-binding protein [76].

#### 2.3.4. Mixed Enzyme

PLA2s have been recruited to venom function by almost all venomous animals [77], acting as a multifunctional toxin with myotoxic, neurotoxic, and hemotoxic activity. We found PLA2s in the transcriptomes of *S. haddoni* (2), *Hs. crispa* (5), and *E. quadricolor* (2).

#### 2.3.5. Pore Forming Toxins

Pore forming toxins are common in cnidarian venoms [78] and disrupt or penetrate cell membranes via lysis [79]. Pore forming toxins that form α-helical barrel structures are referred to as α-PFTs or actinoporins [80]. We recovered actinoporin transcripts in all species except for *S. haddoni.* We found actinoporins in greatest abundance in the heteractine *Hs. crispa* (5). Pore forming toxins that form β-barrel pores are referred to as (β-PFTs) or Membrane Attack Complex/Perforins (MAC/PF) and are found across eukaryotes [81]. As was the case for actinoporins, we recovered transcripts for MAC/PFs in all species except *S. haddoni*, with highest abundance (7) in the heteractine *M. doreensis.*

#### 2.3.6. Allergens and Innate Immunity

Transcripts classified as allergens and innate immunity genes are common in the venom arsenals of arthropod and reptile toxins [82,83] and have been recovered across Cnidaria [50,84]. The stichodactyline *C. adhaesivum* was the only species in this study in which we recover a transcript closely matching snake venom serine protease Dav-PA (3). This protein has fibrinogenolytic, esterolysis, and amidolytic activities [85]. In *E. quadricolor*, we recovered a single transcript closely matching a techylectin-like protein known to lead to platelet aggregation and blood coagulation [53].

Across all transcriptomes we recovered transcripts for the cysteine-rich venom protein, venom allergen 5. Venom serine protease Bi-VSP, a multifunctional enzyme which was previously found in both wasps and in bees [54,55] was also recovered across all species. *Entacmaea quadricolor* was the only species in which we did not recover the silk moth venom serine protease and *Ha. hemprichii* was the only species in which we did not recover transcripts that align to the gene for venom serine carboxypeptidase previously identified in bees and inferred to be involved in the degradation of [86]. We recovered transcripts matching the complement-activating protein *Austrelaps superbus* venom factor 1 [87] in all taxa except *S. haddoni* and *M. doreensis.*

#### 2.3.7. Venom Transcrits of Unknown Function

We find transcripts similar to those associated with venom or toxins in other lineages, but for which precise function or mode of action is unknown. Several of these are part of toxin gene families that are part of physiological pathways and systems other than venom. For example, Endothelial Growth Factors or EGF domain peptides like OMEGA-stichotoxin-Shd4a may have both toxic and EGF activity. We found OMEGA-stichotoxins in all species studied here. We found transcripts matching the sea anemone 8 toxin family [88,89,90] across three species: *C. adhaesivum*, *M. doreensis*, and *E. quadricolor*.

## 3. Discussion

Actiniarians are predatory invertebrates that use venom to catch prey. Previous research on actinarian venoms has focused on describing and characterizing these toxins broadly, and those studies have shown that Actinarian venom composition is largely neurotoxin-rich [3,91] in contrast to the venom of other cnidarians, such as the enzymatically rich toxin arsenal of medusozoans [3,92,93]. Given the presumed defensive function of neurotoxins for their symbiotic clownfish, we expected greater prevalence of neurotoxins in the venom transcriptome of clownfish-hosting sea anemones [20]. Instead, from our findings we conclude that, when looking at the whole venom arsenal reconstructed from transcriptomes, the venom composition of clownfish-hosting sea anemones largely emphasizes predatory rather than defensive behavior.

The prevalence of hemostatic and hemorrhagic transcripts in the expressed venom of clownfish hosting actiniarians is the rationale for interpreting their venom as largely predatory. Within this functional group, we found key gene families which were highly abundant and consistent across all species: for example, transcripts that match platelet aggregation disruptors veficolin-1 and ryncolin were found in high numbers across all species, as were multiple members of the subfamily of venom prothrombin activators. The ecology of clownfish-hosting anemones is likely relevant to understanding their venom [94] suggests that the hemostatic and hemorrhagic “predatory” venom is dual function and important in defense of the fish. Dual function for predatory venom is common [94] and is likely relevant here because the sea anemones themselves are expected to rely minimally on predation, using zooxanthellae to meet most of their nutrition [20]. Future studies of the target and effect of these hemostatic and hemorrhagic venoms should evaluate whether it is more attuned to the diet of the sea anemone host or the predators of the clownfish. 

The prevalence of hemostatic and hemorrhagic transcripts in the expressed venom of clownfish hosting actiniarians is the rationale for interpreting their venom as largely predatory. Within this functional group, we found key gene families which were highly abundant and consistent across all species: for example, transcripts that match platelet aggregation disruptors veficolin-1 and ryncolin were found in high numbers across all species, as were multiple members of the subfamily of venom prothrombin activators. The ecology of clownfish-hosting anemones is likely relevant to understanding their venom [94] and may explain differences from related but ecologically dissimilar species. Dual function for predatory venom is common [94], and hemolytic and hemorrhagic toxins may be important in the defense of the fish. Venom from a species which is invoved in complex evolutionary interactions is likely to have its venom composition shaped by those interactions [1,48]. Sea anemones are generalist predators and thus the breadth of predators of clownfish, rather than the breadth of the host anemone’s diet, would be expected to shape the venom in these species.

The dynamic interactions which occur between clownfish and their venomous hosts places clownfish-hosting sea anemones in a unique category of venomous animals with pharmaceutical potential [62,95]. Among sea anemone-derived toxins, one (a ShK derivative called dalazatide) has passed initial pharmaceutical testing and is currently being used in human trials to combat multiple sclerosis and autoimmune disorders [96,97]. Given the long evolutionary history of Cnidaria and symbiotic association with potential vertebrate targets, clownfish-hosting anemones present a unique system in which toxins with pharmaceutical potential have undergone multualistic co-evolution with vertebrates, rather than co-evolving as predators or prey. Although it is unlikely every putative toxin gene identified here is used in the sea anemone venom arsenal, these accounts of the putative toxin repertore of clownshigh-hosting anemones serve as a starting point to identify additional toxin candidates with pharmaceutical potential.

Beyond what has been previously characterized in these species, we found eight genes affiliated with toxins that have not been reported in actiniarians. These span functional groups and include members of gene families previously reported in cnidarians and those known only from other venomous lineages. The insulin-like venom proteins 302 and 59.1 that we identify in the transcriptome of *C. adhaesivum* and *Ha. hemprichii*, respectively, have not been reported in any other actiniarians, but have been found in other cnidarians [51,98,99,100]. Insulin-like peptides reported from the actiniarians *Oulactis* sp., *N. vectensis,* and *Ex. pallida* [101,102] are cono-insulins either matching to human insulins or cone snail toxins; the relationships between these cono-insulin-like peptides and insulin-like venom proteins 302 or 59.1 are unknown. We did not recover any cono-insulin-like peptides using our pipeline. Like the insulin-like peptides, reticulocalbin is known in cnidarians [98] but not in actiniarians. In contrast, the neurotoxins omega-theraphotoxin-Pm1a and psalmotoxin-1, originally described in tarantulas, have not been reported in any cnidariana. Although hemotoxins were diverse and abundant in the transcriptomes of all taxa (Table 3), snake venom phosphodiesterase was recovered only in *Hs. crispa* and snake venom serine protease (SVSP) Dav-PA was recovered only in *C. adhaesivum*.

No toxin transcript previously reported in anemones was uniquely identified in any of the study species, although some of the more diverse toxins were present in low copy number, occured in only a couple of taxa, or were restricted to a clade (Table 3). Based on the diversity of toxins reported to date for actiniarians, we expect to find Type 1, 2, and 3 sodium channel toxins; types 1, 3, 4, and 5 potassium channel toxins; cytolysin 1 and 3; NEPs; SCRiPS; acrorhagins; and actinoporins (see [4]). The fact that we did not recover all of these in all taxa (Table 3) indicates that transcripts of those genes are either not expressed or expressed at very low levels when the data were collected, or that they are absent from the venom of these species altogether. Of the types expected to be present, acrorhagins are an interesting omission: although these toxins can kill crustaceans [103], they are associated with intraspecific aggression in actinioideans and metridiodeans by virtue of their pattern of expression in tissues involved in self/not self-interactions [104,105,106] and so are expected to be part of the shared repertoire of both clades [4]. However, no clownfish-hosting anemone has structures associated with the use of acrorhagins in other taxa, and none are known to engage in intraspecific competitive interactions. Thus, the lack of expression may be expected based on function, if not on phylogeny. More information about the function of these toxins and a genomic perspective on acrorhagins in species that do and do not engage in intraspecific competition is needed to understand the evolution of these toxin types.

Species-specificity of a toxin implies either a related lineage loss or pseudogenization, or an independent recruitment event involving a protein gaining a toxic function. Because each transcriptome is a snapshot in the expression profile, absence may simply reflect the developmental or physiological state of the animal sampled and should be contextualized with an understanding of how these factors impact venome [12,107,108,109,110]. Although our use of a standard method for assembly, search, and identification of putative venom transcripts mitigates some problems with comparing venom across transcriptomes, given the lack of standardization and replication of transcriptome generation, absent toxins should be interpreted cautiously [111].However, unique occurrences and absences are provocative, indicating novelties that can (and should) be searched for deliberately in subsequent transcriptomic or proteomic studies.

Although venom is dynamic and expression can change across time, space, and clade, we are encouraged and intrigued to see stability across functional groups in lineages that share similar ecologies. All studied transcriptomes were dominated by transcripts matching those known to have hemostatic and hemorrhagic function. Consequently, we infer that this functional group is of primary importance in these species. The fraction of toxins identified as hemostatic or hemorrhagic per transcriptome ranged from 46–66% of total toxins recovered across our study species. These were the most diverse type of toxin in terms of the number of toxin families (Table 3). The second-most prevalent toxin type varies by clade, auxiliary toxins second in Heteractina and neurotoxins being second in both stychodactylines and in Entacmaea. *Macrodactyla doreensis* showed a relatively high abundance of pore forming toxins, consistent with Ashwood et al. [45], which may indicate the particular importance of pore forming toxins in the venom arsenal of *M. doreensis.*

A few published accounts can help contextualize what we report for the clownfish-hosting anemones. In cerithiarians [98], hemostatic and hemorrhagic toxins are the dominant constituents. In *Nematostella* [110] and in *Anthopleura elegantissima* [105], neurotoxins are the most abundant reported toxin, and potentially the most abundant among ancestral actinarians [110]. The proteome of mucus of envenomating *Anthopleura dowii* is also especially rich in neurotoxins, although its tentacle transcriptome is relatively enriched in hemorrhagic toxins (constituting >50% of the reported toxins) [112], which may be due to differences in molecular stability, tissue specific expression (with mucus representing a pooled sample), or individual-level differences in physiological state and expression at any point in time. In *N. vectensis*, the voltage gated neurotoxins are made in the ectodermal gland cells whereas the hemorrhagic toxin metalloprotease is made in the nematocyte [73]. This offers a possible explanation for and the relative abundance and control of abundance for various kinds of toxins, since ectodermal gland cells and nematocytes occur in different densities and arrangements in different parts of the animal.

Transcriptome assembly and completeness is a major determinant of the venom repertoire that can be inferred [111,113]. Because of differences in assembly parameters and growth of databases against which the transcriptomes are queried, we expect to recover transcripts that could not be interpreted or compared in earlier assemblies (Table 1). Furthermore, methodological differences in transcriptome queries make it hard to compare results across studies. Nonetheless, despite differences in assembly and match criteria, our results are broadly consistent with previous studies of cnidarian venoms in our recovery of an array of transcripts associated with the types of venoms found in actiniarians: auxiliary, pore forming, mixed enzyme, neurotoxins, protease inhibitor activities and anemone toxins with unknown functions. In addition to the categories typically used for describing actiniarian venom, we identified allergens and innate immunity toxins and hemostatic and hemorrhagic activity toxins to better reflect functionality of venom in these species.

In general, across the focal taxa, we find that most putative toxin groups are represented by a small number of transcripts, so a greater number of transcripts in the assembly broadly translates into a greater number of gene families. Increasing the number of taxa studied within a lineage did not necessarily increase the perceived venom diversity or the number of toxin families reported for that lineage (Table 2). For example, in the single representative of Entacmaea (*E. quadricolor*), we recovered more putative toxins and gene families than in the two species of Heteractina combined. Although the number of actiniarian-specific toxins varies considerably, the number of actiniarian-specific gene families varies relatively little across samples (range is 12–13, see Table 2). The overall similarity in the percentage of the recovered toxins that belong to actiniarian-specific gene families obscures diversity across taxa in terms of which gene families are present. Furthermore, the proportion of actiniarian-specific transcripts is wide-ranging, with more than double the proportion of actiniarian-specific transcripts in *M. doreensis* than in *E. quadricolor* or in *Ha. hemprichii*.

To properly evaluate the pharmaceutical potential for sea anemones that host vertebrate symbionts, it is important to consider all possible toxin candidates found within a transcriptome or proteome. Further exploration is needed with to properly evaluate the medical potential with with these understudied proteins, which have been shaped by evolution over millions of years.

## 4. Materials and Methods

We analyzed transcriptomes of six species of sea anemones from NCBI (Table 1) using all available data, including transcriptomes from specific tissues rather than the whole body (Table S1). We compiled transcriptomes for *Cryptodendrum adhaesivum* from run IDs SRR14115232, SRR14115233; *Stichodactyla haddoni* from the run Id SRR5397293, *Heterodactyla hemprichii* from run IDs SRR14115226, SRR14115227, SRR14115228, for *Heteractis crispa* from run IDI SRR1950632, SRR1950633, SRR1950656; for SRR14115229; for *Macrodactyla doreensis* for run IDs SRR14115222, SRR14115223, SRR14115224, SRR14115225; and for *Entacmaea quadricolor* from run IDs ERR2045166, ERR2045167, ERR2045168, RR2045169, ERR2045170, EERR204517. Transcriptome construction followed a de novo assembly using Trinity v2.2 [114] using the trimmomatic option, on the Ohio State Supercomputer Center (OSC) running on 2 nodes with 30 CPU each. Transcriptome completeness was determined via BUSCO v5.3.1 [115] against the metazoa_odb10 lineage dataset for each transcriptome. Each transcriptome was bioinformatically annotated for putative venom transcripts. Results per species were then pooled for each species where there were multiple transcriptomes.

Protein-coding regions were predicted from assembled transcriptomes using TransDecoder v5.5.0 (https://transdecoder.github.io, accessed on November 2021), with a minimum sequence length of 50 amino acids. All transcriptomes were queried against the Tox-prot animal venom database using the key word (“Cnidaria,” downloaded November 2021) and the NCBI Protein Database using the key words (“Cnidaria AND ((Toxin) OR (Venom)),” downloaded 23 November 2021) by using blastp from NCBI BLAST + v.2.8.1 [116] using an e-value cutoff of 0.001. Predicted protein-coding region were searched using hmmscan with an e-value cutoff of 0.001 from HMMER 3.1b2 [117] against hidden markov model (HMM) profiles alignments of all the cnidarian venom protein classes from VenomZone (venomzone.expasy.org, accessed on November 2021). The results from all three queries (ToxProt, cnidaria specific-NCBI, and hmmsearch) were combined for each species and only complete coding sequences used for downstream analysis.

Because venoms are secreted proteins and peptides, signal peptides were predicted from the sequences of the combined queries using the SignalP v5.0 server (https://services.healthtech.dtu.dk/service.php?SignalP-5.0, accessed on 23 November 2021) [118]. Sequences with a 70% >probability of having a signaling region were clustered using CD-HIT v.4.6.8 [119] with a cutoff of 0.95. Only the top hit from each cluster was used in downstream analysis. To ensure completeness of venom recovery, sequences which passed these thresholds were used for a reciprocal search against a concatenated transcriptome form each species. The resulting datasets (signal peptide present, with redundant sequences removed) were screened via Blastp with an e-value cutoff of 1 × 10^−5^ against Tox-Prot animal venom database and the NCBI non-redundant protein sequences database (NR DB) (downloaded March 2022), and via hmmsearch (PFAM) search with an evalue cutoff of 1 × 10^−5^ against Pfam (downloaded March 2022). Manual curation of query hits was performed to confirm that annotations from ToxProt and NR DB matched the venom domain from Pfam. The final list of Toxin candidates for each species were classified into protein families and grouped by toxin function. Pipeline can be visualized in Figure S1 (Supplementary Material).

Select venoms were further evaluated by creating alignments using MUSCLE [120] on the EMBL-EBI web server [121] (https://www.ebi.ac.uk/Tools/msa/muscle/, accessed on 23 April 2022) aligning to proteins found on UniProt [122]. Alignment visualization was created on Geneious Prime v2022.2.2 (https://www.geneious.com, accessed on 23 April 2022) (Figures S2–S5).

## Figures and Tables

**Table 1:** Assembly metrics for transcriptome assemblies. The values given in the original assemblies are italicized and in parentheses, dash (-) indicates previously missing metric.

Clade	Taxon [Source]	Transcripts	Genes	N50	BUSCO
Stichodactylina	*C. adhaesivum* [45]	905,882 (628,469)	484,240 (451,132)	1308 (609)	96.2 (93.9)
	*S. haddoni* [46]	196,433 (1,800,000)	150,054 (269,628)	1038 (-)	83.6 (-)
	*Ha. hemprichii* [45]	600,056 (101,150)	165,714 (74,496)	1055 (1370)	94.5 (92)
Heteractina	*Hs. crispa* [47]	655,116 (-)	581,957 (-)	545 (-)	91.8 (-)
	*M. doreensis* [45]	624,291 (-)	218,128 (-)	1328 (-)	91.4 (-)
Entacmaea	*E. quadricolor* [PRJEB21970]	1,198,081 (-)	296,968 (-)	1088 (-)	98.3 (-)

**Table 2:** Number of toxin hits across focal taxa and across families (in bold). (T = number of putative toxins; TF = Number of Toxin Families; AT = Number of Actiniarian Toxins; ATF = Number of Actiniarian Toxin Families).

	T	TF	AT	ATF
*C. adhaesivum*	256	34	88	13
*S. haddoni*	112	33	46	12
*Ha. hemprichii*	115	32	27	13
**Stychodactylina**	**483**	**47**	**161**	**18**
*M. doreensis*	135	25	56	12
*Hs. crispa*	175	35	59	12
**Heteractina**	**310**	**37**	**115**	**13**
*Entacmaea quadricolor* **(Entacmaea)**	**328**	**37**	**79**	**13**
Total	1121	51	355	18

**Table 3:** Putative toxin diversity among focal taxa sorted by various venom categories.

Venom Category	Species	Venom ID	CA	SH	HH	MD	HC	EQ
Auxiliary	sea anemone	Astacin-like metalloprotease toxin M12A	15	5	2	15	13	22
		Nematocyst expressed protein 6	24	8	4	9	13	21
	snake	Cystatin-2	-	2	-	1	1	1
	scorpion	Venom protein 302	1	-	-	-	-	-
		Venom protein 59.1	-	1	-	-	-	-
Pore Forming	sea anemone	Actinoporin family	1	-	1	1	5	2
		MAC/PF	1	-	1	7	1	1
Neurotoxin	sea anemone	Cnidaria small cysteine-rich protein (SCRiP) family	-	-	1	-	-	1
		Cysteine-rich venom protein	8	-	-	2	5	-
		Neurotoxin ShK-like1	-	-	1	-	-	-
		Sea anemone short toxin (type III) family (Delta-actitoxin-Avd2b1)	-	1	1	-	-	-
		Sea anemone structural class 9a	1	1	-	-	-	3
		Sea anemone type 1 potassium channel toxin subfamily	4	3	-	1	1	1
		Sea anemone type 3 potassium channel toxin subfamily (BDS)	7	8	6	1	5	4
		sodium channel toxin Type I	-	3	-	2	2	-
		sodium channel toxin Type II	3	2	6	-	-	-
	sea snail	Conopeptide P-like superfamily	10	2	3	2	5	8
	spider	Omega-theraphotoxin-Pm1a, Neurotoxin 10	-	1	-	-	-	-
		Psalmotoxin-1 (PcTx1)	-	1	-	-	-	-
	centipede	Scoloptoxin	-	1	-	-	2	-
Mixed Enzyme	sea anemone	Phospholipase A2	-	2	-	-	5	2
Protease Inhibitor	sea anemone	Venom Kunitz-type family	7	3	3	9	2	7
Allergen and Innate Immunity	snake	*A. superbus* venom factor 1	2	-	2	-	1	3
		snake venom serine protease Dav-PA	3	-	-	-	-	-
		Snake venom serine protease salmobin	-	-	-	2	1	-
		Venom serine carboxypeptidase	8	2	-	6	7	5
	spider	Techylectin-like protein	-	-	-	-	-	1
	ant	Venom allergen 5	2	3	1	3	6	5
	moth	Venom serine protease	7	1	3	2	1	-
	bee	Venom serine protease Bi-VSP	7	1	3	2	1	3
Hemostatic and Hemorrhagic Toxin	shrew	Blarina toxin	13	1	4	-	3	9
	snake	Coagulation factor V	-	-	1	-	3	1
		Coagulation factor X-activating enzyme	4	1	-	-	3	4
		Cobra venom factor	-	1	2	-	-	1
		Ryncolin	14	8	13	3	3	35
		Snaclec family	7	7	1	10	13	6
		True venom lectin family	4	3	-	5	3	26
		Veficolin-1	17	9	14	9	10	45
		Venom factor (VF)	3	-	4	-	-	1
		Venom phosphodiesterase	-	-		-	1	-
		Venom prothrombin activator	25	5	15	8	32	48
		Zinc metalloproteinase/disintegrin-like (M12B)	7	2	6	6	3	9
	fish	Galactose-specific lectin nattectin	8	6	-	6	1	4
		Natterin-4	-	-	-	-	4	1
	lizard	Venom protease	6	4	5	11	4	12
	spider	Venom peptide isomerase heavy chain	17	5	6	3	7	15
unknown	sea anemone	EGF domain peptide family	6	3	1	2	1	5
		Sea anemone 8 toxin family	1	-	-	1	-	1

## Data Availability

Not applicable.

## Supplementary Materials

The following supporting information can be downloaded at: https://www.mdpi.com/article/10.3390/md20120730/s1, Figure S1: Bioinformatic pipeline for the annotation of venom-like genes, Figures S2–S5: alignments of: NaTx1&2, SHk-like-1, venom 302, sea anemone short type 3 toxins to their gene families, Table S1: Individual transcriptome trinity statistics and BUSCO scores, Table S2: List of Identified venom transcripts (Excel).
