# Supplementary material for: Never, Ever Make an Enemy… Out of an Anemone: Transcriptomic Comparison of Clownfish Hosting Sea Anemone Venoms
Source: Mar Drugs. 2022 Nov 23;20(12):730. doi: 10.3390/md20120730 (PMC9782873; doi:10.3390/md20120730)
Supplement: Supplementary file 1 [file marinedrugs-20-00730-s001.zip › S_FIGURE.pdf]

Transcriptome assembly	-Downloadsampling using SRA<br>-FA STQC<br>-De-novoassembly, Trinityv2.2<br>Trimmomaticoption<br>-BUSCO v5.3.1<br>Metazoa_odb10 lineagedataset<br>-TransDecoder v5.5.0<br>Minimum seq length of 50 amino acids
Candidate toxin identification	-Blast+2.8.1 (e-value 0.001)<br>-Tox-prot animal venom database, keywords ("Cnidaria"), downloaded 2021<br>-NCBI Protein database, keywords "(Cnidaria AND ((Toxin) OR (Venom))"<br>-HMMER 3.1b2<br>-Using all Cnidarian venom protein classes from VenomZone.
Filtering data	-Signal P V5.0 (70%>)<br>-https://services.healthtech.dtu.dk/service.php?SignalP-5.0<br>-CD-HIT v.4.6.8 (cutoff 0.95)<br>-https://sites.google.com/view/cd-hit<br>-Reciprocal blastsearch against a concatenatedtranscriptome
Annotation & Confirmation	-Blastp<br>-Tox-Prot Animal venom database (e-value1e-5)<br>-NCBI non-redundant protein database (NR DB)<br>-Hmmsearch PFAM (e-value 1e-5)<br>- Manual cureation to ensureall resultsmatched<br>-Assigned venom categories basedon uniprot/prior reseach<br>-Create aligmentof select toxins

Supplementary figure s1. Annotation pipeline for project.

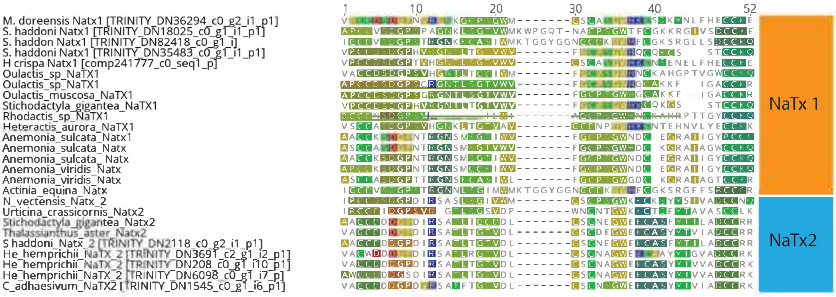

Supplementary figure s2. Alignment shows both NaTx1 (aqua) and NaTx2 (orange) across sea anemone species. All species show a conserved cysteine pattern across both NaTx types. NaTx2 shows distinction with havening a conserved region across amino acids 39-44.

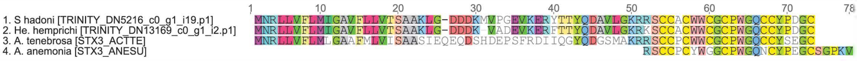

Supplementary figure s3. Alignment showing Sea anemone short type 3 toxins. Mature region shows a rich cysteine composition (CC C-C C-CC C).

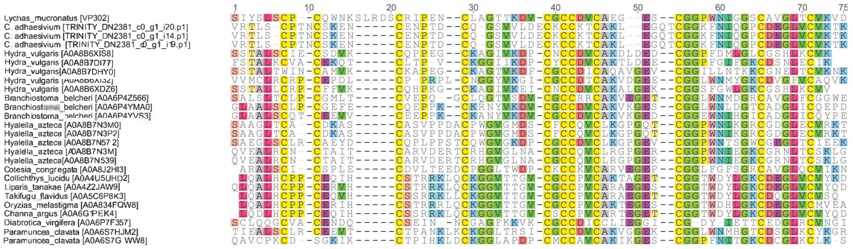

Supplementary figure s4. Alignment showing the insulin like toxin Venom protein 302 across all known species. Alignment shows conserved and rich cystine region (CX5-CC CX4).

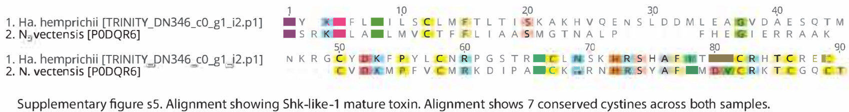

Supplementary figure s5. Alignment showing Shk-like-1 mature toxin. Alignment shows 7 conserved cystines across both samples.
