# Supplementary material for: Never, Ever Make an Enemy… Out of an Anemone: Transcriptomic Comparison of Clownfish Hosting Sea Anemone Venoms
Source: Mar Drugs. 2022 Nov 23;20(12):730. doi: 10.3390/md20120730 (PMC9782873; doi:10.3390/md20120730)
Supplement: Supplementary file 1 [file marinedrugs-20-00730-s001.zip › TABLE_S1.pdf]

Clade	Taxon	Transcripts	genes	N50	Average contig length	Median contig length	percent G/C	BUSCO % (protein)	BUSCO %
Stichodactylina	*C.adhaesivum (body)*	634715	423320	1127	724.23	403	39.86	83.5	87.2
	C.adhaesivum (nematocyst)	271167	160450	1574	962.67	571	46.2	87.8	91.1
	S.haddoni	196433	150054	1038	718.72	449	43.48	81.2	83.7
	Ha.hemprichii (A)	131444	116964	605	507.94	339	46.63	50.2	50.1
	Ha.hemprichii (B)	161588	132154	1352	834.03	479	47.14	85.8	86.5
	Ha.hemprichii (C)	140865	123392	870	628.95	401	48.63	60.4	59.7
	Ha.hemprichii (D)	166159	139624	1232	768.72	430	45.9	92.2	92.5
Heteractina	*Hs.crispa (combined)*	655116	581957	545	459.17	263	45.17	91.8	92.8
	M.doreensis (1)	169310	125063	1280	829.65	518	45.69	81.7	82.6
	M.doreensis (2)	128634	102820	678	633.31	388	45.79	49.8	50.1
	M.doreensis (3)	174026	123083	1609	984.01	587	46.27	85.5	87.2
	M.doreensis (4)	152321	113560	1326	856.41	549	47.49	70.7	75
Entacmaea	*E.quadricolor (1)*	159879	110724	1398	815.42	433	38.95	95.1	95.8
	E.quadricolor (2)	182027	136593	1001	687.55	407	40.25	89.3	90.1
	E.quadricolor (3)	218132	166239	1051	722.73	438	41.93	90.7	92.4
	E.quadricolor (4)	211617	161980	1058	728.22	448	42.49	93	94
	E.quadricolor (5)	216944	166660	1054	713.1	415	41.54	94.2	94.8
	E.quadricolor (6)	209482	159611	1052	722.63	436	41.8	92.6	93.8

Supplementary table S1. The the table shos individual transcriptomes state for each SRA sample. Results were sourced by using Trinity_stats.pl within Trinity. BUSCO v5.4.2 was used to retrive BUSCO scores.
